# 4R tau drives endolysosomal and autophagy dysfunction in frontotemporal dementia

**DOI:** 10.1080/15548627.2023.2300917

**Published:** 2024-01-04

**Authors:** Christy Hung, Rickie Patani

**Affiliations:** a Human Stem Cells and Neurodegeneration Laboratory, The Francis Crick Institute, London, UK; b UCL Great Ormond Street Institute of Child Health, Zayed Centre for Research into Rare Disease in Children, London, UK; c Department of Neuromuscular Diseases, Queen Square Institute of Neurology, University College London, London, UK

**Keywords:** Autophagy, endosomes, lysosome, tau, tauopathy

## Abstract

Dysfunction of the neuronal endolysosome and macroautophagy/autophagy pathway is emerging as an important pathogenic mechanism in frontotemporal dementia (FTD) and amyotrophic lateral sclerosis (ALS). The *VCP* (valosin-containing protein) gene is of significant relevance, directly implicated in both FTD and ALS. In our recent study, we used patient-derived stem cells to study the effects of *VCP* mutations on the endolysosome and autophagy system in human cortical excitatory neurons. We found that *VCP* mutations cause an abnormal accumulation of enlarged endosomes and lysosomes, accompanied by reduced autophagy flux. *VCP* mutations also lead to the spatial dissociation of intra-nuclear RNA-binding proteins, FUS and SFPQ, which correlates with alternative splicing of the *MAPT* pre-mRNA and increased tau phosphorylation. Importantly, we found that an increase in the 4R-tau isoform is sufficient to drive toxic changes in healthy human cortical excitatory neurons, including tau hyperphosphorylation, endolysosomal dysfunction, lysosomal membrane rupture, endoplasmic reticulum stress, and apoptosis. Together, our data suggest that endolysosomal and autophagy dysfunction could represent a convergent pathogenic “design principle” shared by both FTD and ALS.

Amyotrophic lateral sclerosis and frontotemporal dementia are two devastating and untreatable neurodegenerative diseases that exist along a spectrum of clinical, genetic, and pathological features. The *VCP* gene is relevant here, being associated with both FTD and ALS. The displacement of canonical RNA binding proteins (RBPs) like FUS (FUS RNA binding protein), TDP-43 (TAR DNA binding protein), and SFPQ (splicing factor proline and glutamine rich) from the nucleus, along with tau, represents pathological hallmarks in both FTD and ALS. However, the precise molecular mechanisms by which *VCP* mutations affect the endolysosomal pathway, disrupt RBP interactions and splicing function and subsequently influence tau pathology, remain poorly understood.

In our recent study [1], we systematically investigated the effects of FTD-causing *VCP* mutations on neuronal homeostasis using a highly enriched and functionally validated human induced pluripotent stem cell (hiPSC)-derived, patient-specific cortical neuron model. Using high-resolution instant structured illumination microscopy to measure the size of endogenous endosomes and lysosomes, we observed that familial *VCP* mutations lead to the abnormal accumulation of enlarged endosomes and lysosomes. Measuring autophagic flux through a well-established quantitative western-blot approach, we determined that the degradative phase of autophagy is compromised in human *VCP* mutant cortical neurons. Additionally, we demonstrated that the aberrant interaction between FUS and SFPQ in human *VCP* mutant cortical neurons, correlates with dysregulation of alternative splicing of *MAPT* pre-mRNA and increases tau phosphorylation. Finally, we established that endolysosomal dysfunction, lysosomal membrane rupture, endoplasmic reticulum stress and apoptosis are induced by elevated levels of 4R tau, as these defects are recapitulated using a splice-switching antisense oligonucleotide to increase 4R tau levels in healthy human neurons. This raises the prospect of designing therapeutics to reduce 4R tau levels in order to delay disease initiation or to slow down progression.

Dysfunction in the endolysosomal pathway is increasingly recognized as a significant pathogenic process in both FTD and ALS. Several genes associated with these conditions, including *CHMP2B*, *GRN*, and *TMEM106B*, affect the endolysosomal system. Notably, a recent study highlighted the convergence of two FTD-ALS genes, *C9orf72* and *TBK1*, on the endolysosomal pathway, leading to TDP-43 pathology and degeneration. Our data strongly suggest that activating the lysosomal and autophagy pathways holds promise as a therapeutic strategy for FTD and ALS. However, critical questions remain unanswered regarding its specific role in disease pathogenesis. For instance, do pathological variants in these genes exert their pathogenic effects in a cell-autonomous manner, or is it non-cell autonomous? While the lysosomal system has been extensively studied in human neurons, the potential consequences of lysosomal dysfunction in glial cells, such as astrocytes and microglia, remain incompletely understood. Specifically, what is the relative contribution of the neuronal endolysosomal pathway versus the glial endolysosomal pathway to neuropathology in neurodegenerative diseases? Additionally, we must consider how an upregulation of autophagy might disrupt the delicate balance between autophagosome formation and lysosomal degradation. Based on our recent findings, we propose that reducing the levels of the 4R tau isoform in the endolysosomal pathway through siRNA or antisense oligonucleotides, or employing a 4R to 3R splice switching antisense oligonucleotide strategy in the early disease stages, may represent an effective therapeutic approach to mitigate autophagic defects in FTD-ALS. Overall, our study suggests that elevated 4R tau is sufficient to drive dysfunction of the endolysosomal-autophagic system. As such, this work establishes a foundation for future investigation into cellular pathways enhancing autophagy and lysosomal proteolytic activity as an approach to ameliorating neurodegeneration in FTD and ALS. Future work will focus on the precise mechanisms by which elevated 4R tau disrupts cellular homeostasis but also to test the hypothesis that mislocalized RBPs work together to disrupt endolysosomal integrity.
